# Whole genome profiling of short-term hypoxia induced genes and identification of HIF-1 binding sites provide insights into HIF-1 function in *Caenorhabditis elegans*

**DOI:** 10.1101/2023.11.15.567310

**Published:** 2023-11-17

**Authors:** Dingxia Feng, Long Qu

**Affiliations:** 1 Department of Genetics, Development and Cell Biology, Iowa State University, Ames, Iowa, United States of America; 2 Department of Statistics, Iowa State University, Ames, Iowa, United States of America

## Abstract

Oxygen is essential to all the aerobic organisms. However, during normal development, disease and homeostasis, organisms are often challenged by hypoxia (oxygen deprivation). Hypoxia-inducible transcription factors (HIFs) are master regulators of hypoxia response and are evolutionarily conserved in metazoans. The homolog of HIF in the genetic model organism *C. elegans* is HIF-1. In this study, we aimed to understand short-term hypoxia response and to identify HIF-1 direct targets in *C. elegans*. The central research questions were: (1) which genes are differentially expressed in response to short-term hypoxia? (2) Which of these changes in gene expression are dependent upon HIF-1 function? (3) How do HIF-1-dependent hypoxia-responsive genes affect hypoxia adaptation? (4) Which genes are the direct targets of HIF-1? We combine whole genome gene expression analyses and chromatin immunoprecipitation sequencing (ChIP-seq) experiments to address these questions. In agreement with other published studies, we report that HIF-1-dependent hypoxia-responsive genes are involved in metabolism, oxidation-reduction process, and stress response. Some HIF-1-dependent hypoxia-responsive genes like *efk-1* and*phy-2* dramatically impact survival in hypoxic conditions. HIF-1 co-immunoprecipitates with genomic regions proximal genes involved in stress response, protein processing in endoplasmic reticulum, and cell recognition. Further, some of these potential HIF-1 direct targets are differentially expressed under short-term hypoxia or are differentially regulated by mutations that enhance HIF-1 activity.

## Introduction

Oxygen is essential to aerobic organisms for energy production and cellular redox environment maintenance [1]. However, during development, disease and homeostasis, animals are often challenged by oxygen deprivation (hypoxia). In mammals, the majority of transcriptional response to hypoxia is mediated by the hypoxia-inducible transcription factors HIFs. Understanding HIF function and regulation has the potential therapeutic significance in common hypoxia-related diseases, such as cancer, arthritis and ischemia. The heterodimeric HIF complexes consist of α and β subunits, and both subunits are bHLH (basic-helix-loop-helix)-PAS (PER/ARNT/SIM) domain proteins [1, 2]. There are three HIFα and three HIFβ homologs in the human genome [3]. HIFβ has multiple bHLH-PAS dimerization partners and is relatively stable and abundant. In contrast, HIFα is short-lived under well-oxygenated conditions and is dedicated to hypoxia response [4–6]. The stability of mammalian HIFα is regulated by the PHD-VHL pathway. When oxygen levels are high enough, HIFα is hydroxylated by prolyl hydroxylase proteins (PHDs), in reactions that require oxygen as substrate. The hydroxylated HIFα is then targeted by the E3 ligase VHL (von Hippel-Lindau tumor suppressor) for proteasomal degradation.

The nematode *C. elegans* is a powerful genetic model organism for studying HIF function and regulation [7], and its genome encodes single homologs for HIFα and HIFβ, named HIF-1 and AHA-1, respectively. While *hif-1*α −/− mice die by E9.0 with severe vascular defects [8, 9], *C. elegans hif-1*(*ia04*) loss-of-function mutants survive and develop normally in room air but are defective in hypoxia adaption [10–12]. As in mammals, the AHA-1 subunit is stable, while the stability of HIF-1 is regulated by oxygen levels [11, 13, 14]. The PHD-VHL pathway for HIF-1 stability regulation is simplified in *C. elegans*, too. There is only one counterpart for PHD and VHL in *C. elegans*, named EGL-9 and VHL-1, respectively [13]. In addition to its role in targeting HIF-1 for oxygen-dependent degradation, EGL-9 also inhibits HIF-1 activity: HIF-1 targets are expressed at higher levels in *egl-9(sa307)* loss-of-function mutants than in *vhl-1(ok161)* loss-of-function mutants [15, 16].

Prior studies [17, 18] have described oxygen-dependent changes in *C. elegans* gene expression, but the direct targets of HIF-1 are not fully described. Here, we take advantage of *C. elegans egl-9* mutants that fail to degrade HIF-1 and ask which DNA sequences co-immunoprecipitate with HIF-1. To determine which of these targets are likely the immediate targets of HIF-1, we identify the genes that are differentially regulated by short-term (2 hours) hypoxia treatment and ask whether some of the HIF-1 targets are essential to survival in hypoxic conditions.

## Results

### Identification of genes responsive to short-term hypoxia treatment

To define the immediate gene expression changes caused by hypoxia, we treated L4-stage wild-type N2 worms with 0.5% oxygen for 2 hours and compared the whole genome gene expression profile under hypoxia with that in room air. The complete analysis results are provided in S1 Table. Among the 18,011 unique genes assayed, the mRNA levels of 681 genes were changed by hypoxia: 437 genes were up-regulated (S2 Table), and 244 genes were down-regulated (S3 Table). The enriched biological functions for genes up-regulated by short-term hypoxia were oxidation-reduction process, response to stress, transition metal ion binding, small molecule metabolic process, and cellular response to unfolded protein (Table 1). The representative genes for each functional group were listed in Table 1. The full lists of genes for each functional group were provided in S4 Table.

The enriched biological terms for genes down-regulated by short-term hypoxia were single-organism metabolic process, monocarboxylic acid metabolic process, and oxidation-reduction process (Table 2). The representative genes for these functional groups were listed in Table 2. The full lists of genes for these functional groups were provided in S5 Table.

To further validate the data, we referred to genes that had been shown to be hypoxia-responsive in other published studies. RNase protection, RNA blot and real-time qRT-PCR assays had shown that F22B5.4, *nhr-57, fmo-12/fmo-2, egl-9,phy-2, cah-4*, K10H10.2/*cysl-2*, F26A3.4, C12C8.1/*hsp-70, acs-2* and *pck-1* were induced by hypoxia in N2 [17–20]. As expected, the microarray analyses described herein showed that all of these genes were induced by 2 hours of hypoxia in N2 (S2 Table). We also compared this dataset to a prior microarray study that identified 490 genes as hypoxia responsive when L3-stage N2 worms were treated with 4 hours of 0.1% oxygens [18]. While the larval stage and duration of hypoxia treatment were different, we were able to identify 50 genes that exhibited hypoxia-dependent changes in gene expression in these two experiments. The overlap was significant (*p*-value = 2.46E-10, by Fisher’s exact test), suggesting that both experiments identified gene expression changes responding to moderate hypoxia. In sum, the consistency with prior studies supported that our microarray data and analyses were promising.

### HIF-1-dependent short-term hypoxia responses

**Fig 1.**
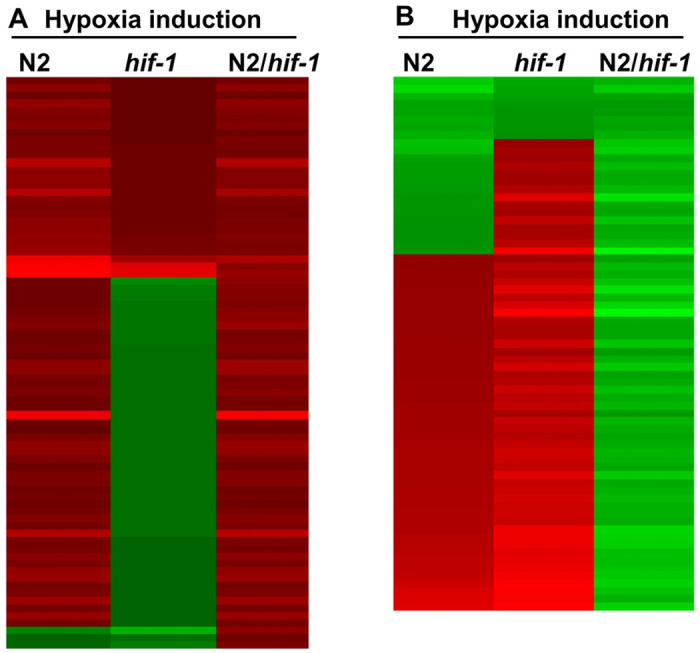
Hypoxia inductions of genes regulated by HIF-1 under hypoxia. (A, B) The heat map illustrations of hypoxia-dependent changes in gene expression for which HIF-1 was a positive regulator (A) or a negative regulator (B). Values < 0 were green, values > 0 were red. The color intensities corresponded to the induction levels in S6 (for Fig 1A) and S7 (for Fig 1B) Tables.

We next asked which short-term hypoxia-responsive gene expression changes were dependent upon HIF-1. To do this, we compared the hypoxia responses in N2 and *hif-1(ia04)* loss-of-function mutants, and this identified 124 genes whose hypoxia responses were different in *hif-1(ia04)* relative to N2 (S6 and S7 Tables). Among these, 64 genes were positively regulated by HIF-1 under hypoxia (their hypoxia inductions were higher in N2 than in *hif-1(ia04)*) (S6 Table), and 60 genes were negatively regulated by HIF-1 under hypoxia (their hypoxia inductions were lower in N2 than in *hif-1(ia04)*) (S7 Table). The heat maps in Fig 1 illustrate their hypoxia inductions in N2 and *hif-1(ia04)*, and the relative inductions (N2/*hif-1(ia04)*).

The enriched biological functions for genes positively regulated by HIF-1 under hypoxia were oxidation-reduction process, metabolic pathways, alpha-amino acid metabolic process, fatty acid metabolic process, and sulfur metabolism (Table 3). Several genes with important functions drew our attention. For example, *cysl-2, ethe-1* and *sqrd*-1 had been shown to have roles in detoxifying hydrogen sulfide (H_2_S) and hydrogen cyanide (HCN) [21], and *sqrd*-1 also maintained translation in H_2_S [22]. *mce-1, mmcm-1*, ZK550.6, *acox-1.6, gbh-2* and*fat-5* had been shown to be involved in lipid metabolism, and *asns-2, cysl*-2 and *ddo-1* had central roles in amino acid metabolism. *pck*-1 was integral to gluconeogenesis [20, 23], and *fmo-1* and *fmo-2* were involved in phase I detoxification. The *phy-2* (prolyl 4-hydroxylase) had been shown to have roles in cuticle collagen synthesis [24]. These findings were well aligned with previously published real-time qRT-PCR experiments that had shown that *pck-1, cysl-2* and *phy-2* were positively-regulated by HIF-1 under hypoxia [20].

The enriched biological functions for genes negatively regulated by HIF-1 under hypoxia were response to stress, and organic acid metabolic process (Table 4). Genes in the response to stress group included F-box A protein genes (*fbxc-58* and *fbxc-60*), infection response gene (*irg-2*), TIR domain protein gene *tir-1*, and UDP-glucuronosyl transferase gene *ugt-44*, and others. Genes in the organic acid metabolic process group included genes for lipid metabolism (*acdh-2, acs-2, elo-2, hacd-1* and *sur-5*), and others (Table 4).

### Genes responsive to both short-term and persistent HIF-1 activities

We anticipated that some of the genes that were responsive to short-term hypoxia would also be differentially expressed in mutants that over-expressed HIF-1 targets. To explore this question further, we compared the findings summarized in S2 and S3 Tables (genes up-regulated or down-regulated by 2-hour hypoxia) with genes that were mis-regulated in the four HIF-1 negative regulator mutants (*vhl-1(ok161), rhy-1(ok1402), egl-9(sa307)*, and *swan-1(ok267);vhl-1(ok161)* double mutants (See the related study entitled “Transcriptome analyses describe the consequences of persistent HIF-1 activation and reveal complementary roles for HIF-1 and DAF-16 in hypoxia survival in *Caenorhabditis elegans*”). This led to the identification of 23 genes were both positively regulated by HIF-1 under short-term hypoxia and up-regulated in all the four HIF-1 negative regulator mutants, in addition to 3 genes were both negatively regulated by HIF-1 under short-term hypoxia and down-regulated in all the four mutants (Tables 5 and 6). The molecular functions of the 23 genes positively regulated by both short-term and persistent HIF-1 activities were diverse, including genes for lipid metabolism (*mce-1, mmcm-1*, ZK550.6 and *gbh-2*), H_2_S and HCN detoxification (*cysl-2, ethe-1* and *sqrd*-1), gluconeogenesis (*pck-1*), and protein synthesis regulation (*efk-1*), as well as collagen synthesis (*phy-2*), and others. The 3 genes negatively regulated by both short-term and persistent HIF-1 activities were T28A11.2 (hypothetical protein), *acdh-2* (Acyl CoA dehydrogenase), and *acs-2* (fatty acid CoA synthetase family).

### Effects of HIF-1-dependent hypoxia-responsive genes on hypoxia adaptation

To further understand HIF-1 function for hypoxia adaptation, we examined the requirement for genes that were induced by hypoxia in a HIF-1-dependent manner, with a focus on genes that had not been tested for their effects on hypoxia survival or had not been shown to have essential roles in development in room air. The actual number of genes tested was further limited by the availability of the mutant and RNAi strains. Twenty-seven mutants or RNAi treatments were examined to test 23 genes, and these genes had functions in multiple biological processes, including lipid metabolism, protein and amino acid metabolism, detoxification and stress response, ion transport, oxygen binding, vitamin biosynthesis, cellular signaling, protein translation regulation and collagen synthesis.

**Fig 2.**
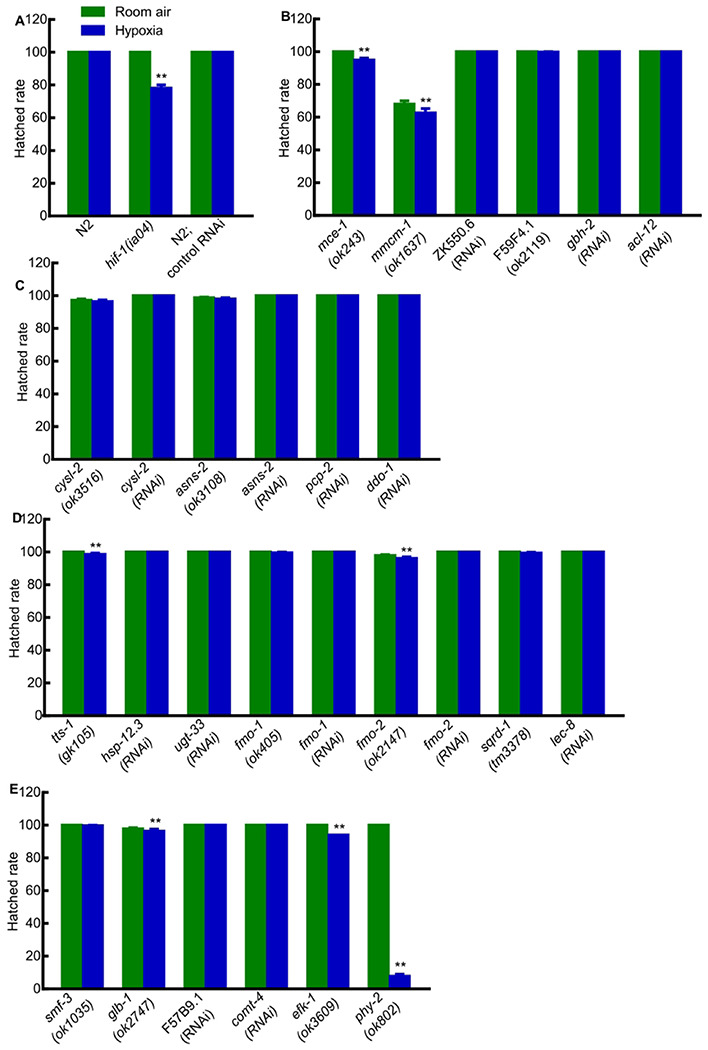
Effects of HIF-1-dependent hypoxia-responsive genes on embryogenesis. (A) Hatched rates of N2, *hif-1(ia04)* and N2;control RNAi in room air and hypoxia. (B-E) Hatched rates in room air and hypoxia for animals lacking specific genes related to (B) lipid metabolism; (C) amino acid metabolism; (D) detoxification and stress response; (E) ion transport (*smf-3*), oxygen binding (*glb*-1), vitamin biosynthesis (F57B9.1), cellular signaling (*comt-4*), protein translation regulation (*efk-1*) and collagen synthesis (*phy-2*). Values were mean ± SEM calculated from three biological replicates. The total number of animals assayed from three biological replicates for each strain in room air or hypoxia ranged from 205 to 661. **p < 0.01.

**Fig 3.**
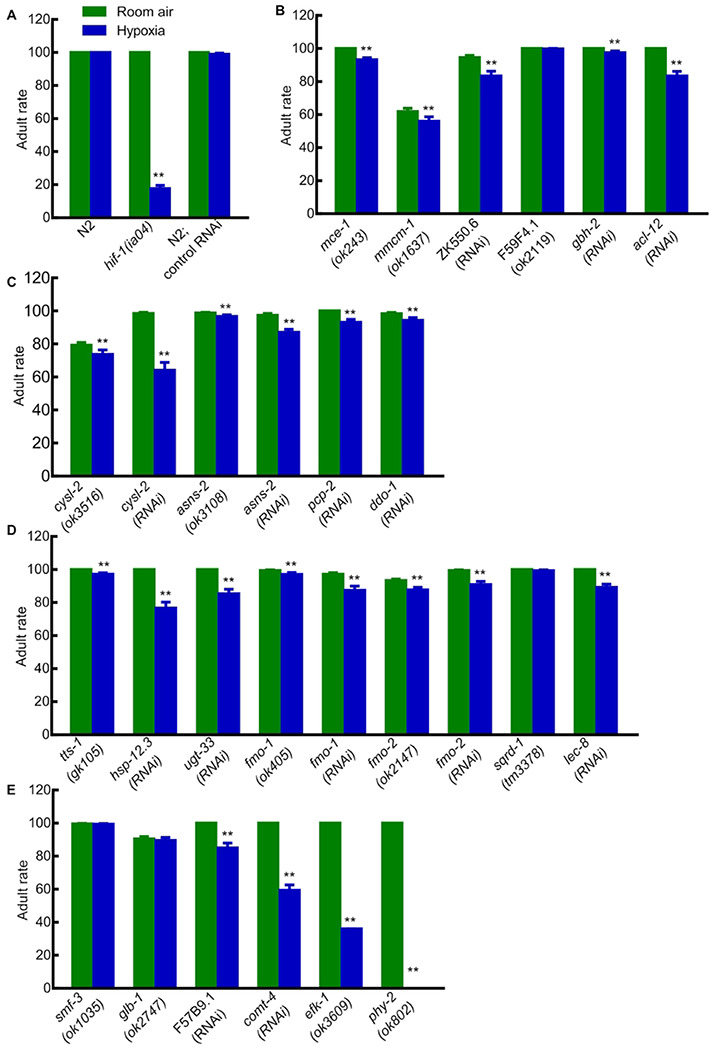
Effects of HIF-1-dependent hypoxia-responsive genes on survival to adulthood. (A) Rates of survival to adulthood for N2, *hif-1(ia04)* and N2;control RNAi in room air and hypoxia. (B-E) Rates of survival to adulthood in room air and hypoxia for animals lacking specific genes related to (B) lipid metabolism; (C) amino acid metabolism; (D) detoxification and stress response; (E) ion transport (*smf-3*), oxygen binding (*glb*-1), vitamin biosynthesis (F57B9.1), cellular signaling (*comt-4*), protein translation regulation (*efk-1*) and collagen synthesis (*phy-2*). Values were mean ± SEM calculated from three biological replicates. The total number of animals assayed from three biological replicates for each strain in room air or hypoxia ranged from 205 to 661. **p < 0.01.

To assay the effects of a particular gene on hypoxia development and survival, we compared the abilities of animals to survive embryogenesis and larval development in hypoxia *versus* room air. These data are illustrated in Figs 2 and 3 (see also S8 Table). Wild-type N2 or N2;control RNAi (feeding L4440 empty vector) and *hif-1(ia04)* mutants were used as controls. As expected, N2 and N2;control RNAi animals were tolerant to hypoxia: their survival rates did not decrease under hypoxia compared to room air (*p*-values > 0.05). By contrast, *hif-1(ia04)* mutants were sensitive to hypoxia: only 78% hatched and 18% survived to adulthood in hypoxic conditions (**p-values < 0.01) (Fig 2, Fig 3 and S8 Table).

Among the 27 mutants or RNAi conditions tested, 7 showed decreases in embryonic viability under hypoxia compared to room air, and 23 showed decreases in larval viability (**p-values < 0.01). The hypoxia effect of HIF-1-depent hypoxia-responsive genes on larval viability was stronger than that on hatched rate. Some mutant animals could hatch under hypoxia, but could not make to adulthood. In agreement with prior studies [17, 18], most of the genes tested had lesser roles in hypoxia survival than *hif-1* (hypoxia treatment decreased their embryonic or larval viability by no more than 20%). Notably, mutations in *hsp-12.3, cysl-2, comt-4, efk-1* or*phy-2* strongly impacted hypoxia survival, reducing survival to adulthood 23%, 34%, 41%, 64% and 100%, respectively, under hypoxia compared to room air.

### Identifying the direct targets of HIF-1 by ChIP-seq

**Fig 4.**
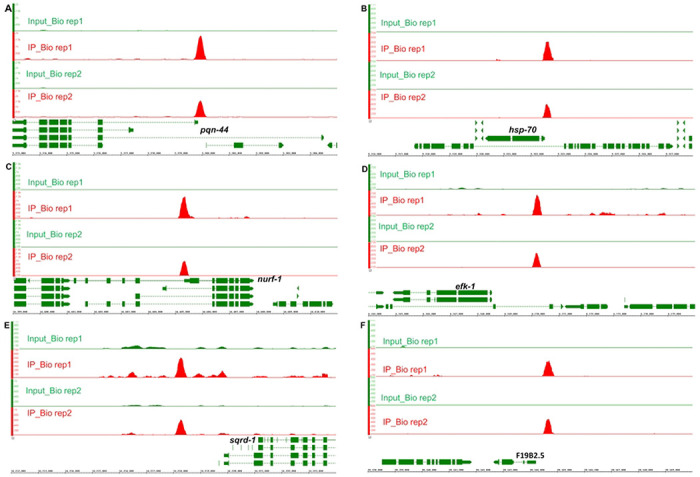
HIF-1 binding regions near six genes regulated by hypoxia or HIF-1. (A) A 600 bp HIF-1 binding region (chrI:5379600-5380199) within *pqn-44* (chrI:5372851-5384508). (B) A 400 bp HIF-1 binding region (chrI:9322400-9322799) upstream of *hsp-70* (chrI:9320325-9322519) and overlap with 5’ UTR. (C) A 600 bp HIF-1 binding region (chrII:14405000-14405599) within *nurf-1* (chrII:14390713-14407911). (D) A 400 bp HIF-1 binding region (chrIII:6170000-6170399) 1453 bp upstream of the transcription start of *efk-1* (chrIII:6164906-6168547). (E) A 600 bp HIF-1 binding region (chrIV:14218000-14218599) 1272 bp upstream of the transcription start of *sqrd-1* (chrIV:14219871-14224440). (F) A 600 bp HIF-1 binding region (chrV:20164400-20164999) 235 bp upstream of the transcription start of F19B2.5 (chrV:20162615-20164165). The images showed the IGB ChIP-seq signals in both biological replicates. For each binding region in each biological replicate, the minimum number for the y-axis scale was the normalized average input tag count, and the maximum number was the averaged ChIP tag count as provided in S9 Table.

**Fig 5.**
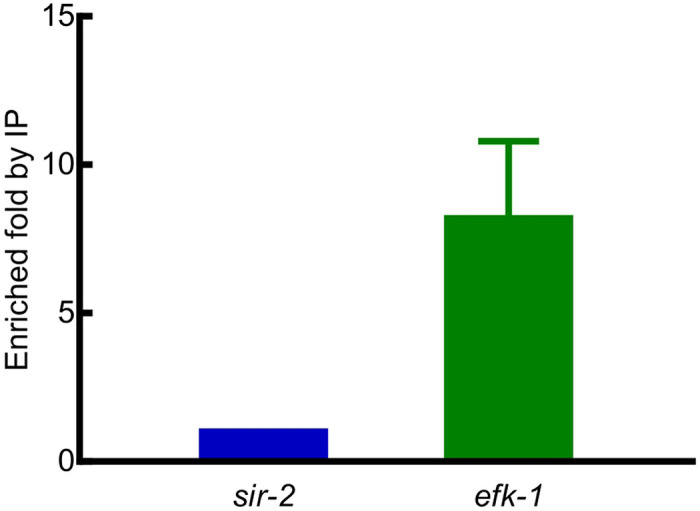
*efk-1* ChIP-qPCR. ChIP-qPCR to verify the HIF-1 binding region in the *efk-1* promoter. The *sir-2* promoter region was used as the reference. Values were mean ± SEM calculated from three biological replicates.

To identify the genome sequences bound by HIF-1, we performed co-immunoprecipitation experiments. While there are six splicing isoforms of *hif-1*, the isoform a (*hif-1a*) had been shown to be essential for longevity and stress resistance [23, 25, 26]. Accordingly, we identified DNA sequences that co-immunoprecipitated with an epitope-tagged version of this HIF-1 isoform. From both biological replicates, we identified 94 reproducible HIF-1 binding peaks (FDR ≤ 0.05 and fold enrichment ≥ 1.6). The summaries of these peaks (including peak coordinates, sizes, distributions, ChIP and input tag counts, fold enrichments, target genes and their expressions under hypoxia and in the HIF-1 negative regulator mutants, et al.) are provided in S9 Table. Sequences co-immunoprecipitated with HIF-1 were provided in S1-S6 Files, organized by chromosomes and peak coordinates, one chromosome one file. ChIP signals were visually verified in IGB (Integrated Genome Browser) and provided in S1–S6 Figs, organized by chromosomes and target genes, one chromosome one file. As representatives, the IGB ChIP signals for *pqn-44* (prion-like-(Q/N-rich)-domain-bearing protein), *hsp-70* (heat shock protein), *nurf-1* (nucleosome remodeling factor complex homolog), *efk-1* (eukaryotic elongation factor 2 kinase), *sqrd-1* (sulfide quinone oxidoreductase) and F19B2.5 (SNF2_N domain-containing protein) were presented in Fig 4. These six genes were among the HIF-1 direct targets whose expressions were changed by hypoxia or in the HIF-1 negative regulator mutants (see below Table 8). HIF-1 bound at different locations relative to these genes. The binding regions for *pqn-44* and *nurf-1* were within the coding regions. The HIF-1 binding site near *hsp-70* was upstream of the transcription start sites and overlapped with 5’ UTRs. The HIF-1 binding sites for *efk-1, sqrd-1* and F19B2.5 were also upstream of the transcription start sites (Fig 4). Among the HIF-1 direct targets whose expressions were changed by hypoxia or in the HIF-1 negative regulator mutants, *efk-1* was of particular interest. As shown in Figs 2 and 3, animals lacking *efk-1* function are less able to survive hypoxia. Gene expression studies show that *efk-1* expression is regulated by hypoxia and HIF-1 (see below Table 8). We verified the HIF-1 binding region in the *efk-1* promoter by ChIP-qPCR. The enrichment of this region was 8-fold relative to the reference *sir-2* promoter region (Fig 5). Consistent with this finding, this region was also identified as a HIF-1 binding site by the ModERN project [27].

**Fig 6.**
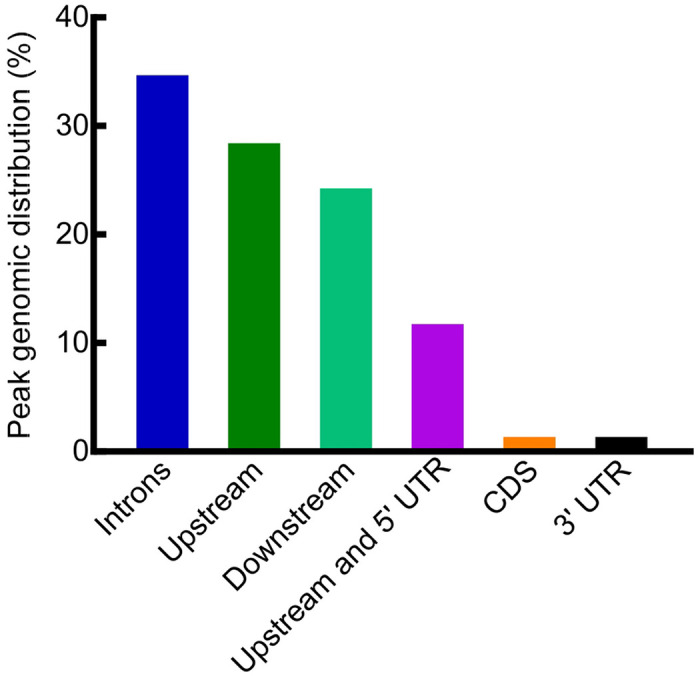
Genomic distributions of HIF-1 ChIP peaks. Genomic distributions of HIF-1 ChIP peaks relative to target genes. The detailed distributions for each peak were provided in S9 Table.

Most of the HIF-1 binding peaks were located in introns (34.38%), upstream of the transcription start sites (28.13%), or downstream of the transcription stop sites (23.96%). The rest of binding peaks lied upstream of the transcription start sites and overlap with 5’ UTRs (11.46%), or in the coding regions (1.04%), as well as in 3’ UTRs (1.04%) (Fig 6 and S9 Table). A majority of the HIF-1 binding peaks (60/94 = 64%) contained sequences similar to mammalian HRE (hypoxia response element) 5’-RCGTG-3’ (R = A or G), or the mandatory core HRE 5’-CGTG-3’ [28] (S9 Table and S1–S6 Files).

These 94 HIF-1 binding regions were annotated to 96 genes (S9 Table). In this study, these genes were treated as HIF-1 direct target genes. The enriched biological terms associated with HIF-1 direct target genes were protein processing in endoplasmic reticulum, response to stress, and cell recognition (Table 7). Genes in the protein processing in endoplasmic reticulum group included heat shock protein genes (*hsp-70, hsp-90* and *dnj-12*), and others. Genes in the response to stress group included heat shock protein genes (*hsp-16.2, hsp-16.41, hsp-70, hsp-90, hsp-110*, and *dnj-12*), *atg-5* (autophagy protein 5), *tir-1* (TIR domain protein), and others. And genes in the cell recognition group included *cua-1* (CU (copper) ATPase), *madd-2* (muscle arm development defective), and others (Table 7).

### Genomic regions that co-immunoprecipitate with HIF-1 map to genes that are differentially regulated by conditions or mutations that alter HIF-1 function

Among the 96 gene regions that co-immunoprecipitated with HIF-1, 24 mapped to genes that were regulated by HIF-1 under short-term hypoxia or in mutants with constitutively active HIF-1 (single mutants of *vhl-1(ok161), rhy-1(ok1402)* and *egl-9(sa307)*, and *swan-1(ok267);vhl-1(ok161)* double mutants) (Table 8).

An interesting feature emerging from the expression patterns of HIF-1 direct targets was that they responded to HIF-1 in differing contexts. Some genes responded to HIF-1 under short-term hypoxia (for example, W03F9.1 and *tir*-1), while others were differentially expressed in mutants in which HIF-1 was constitutively active (for example, *oat-1* and *cpr-3*). As noted in Table 8, some of the genes that coimmuniprecipitaed with HIF-1 had been shown to respond to HIF-1 under both short-term hypoxia and in the mutants with constitutively active HIF-1 (like *efk-1* and *sqrd-1*).

## Discussion

Transcription factors HIFs are the master regulators of hypoxia response. Identification of direct HIF-1 targets is a major step towards more fully understanding the transcriptional networks controlled by *C. elegans* HIF-1. Here, we add to the literature that describes hypoxia-responsive gene expression, in ways that provide new insights to HIF-1 mediated immediate hypoxia response. By cross-referencing the genes that are differentially regulated by hypoxia or HIF-1 with those genomic regions that co-immunoprecipitate with HIF-1, we can describe the direct and downstream targets of HIF-1 with greater confidence.

### Refined understanding of HIF-1 mediated short-term hypoxia response

The transcriptome analyses confirmed and expanded prior studies that have investigated the roles of HIF-1 in hypoxia response. The results of each study is, of course, influenced by the hypoxia regimen, the larval stage tested, and the genetic background. Here, we opted to use multiple strategies to induce HIF-1 through hypoxia (0.5% O2 for 2 hours) or through loss-of-function mutations in HIF-1 negative regulators. By compiling and cross-referencing these data sets, we can develop a list of high-confidence HIF-1 targets. As a complementary experimental strategy, we identified genomic regions that co-immunoprecipitated with HIF-1.

These data sets provide insights to the myriad of hypoxia-responsive functions regulated by HIF-1. Some of these functions are evolutionarily conserved, especially stress response and energy metabolism. There are notable overlaps between genes regulated by HIF-1 under hypoxia with those regulated by DAF-16 [29] (*p*-value = 6.80E-10, by Fisher’s exact test) or with those regulated by H_2_S [30] (*p*-value = 4.04E-05, by Fisher’s exact test), and this suggests that HIF-1 has broad roles in stress response. In the wild, *C. elegans* can live in hypoxic microenvironments, and regulations of stress response pathways are likely to be critical to intergenerational survival [31]. Some of the key findings are summarized in Table 8, and multiple lines of evidence point to key direct targets of HIF-1. These include elongation factor kinase *efk-1*, the heat shock factor *hsp-16.2*, the mitochondrial sulfide quinone oxidoreductase *sqrd-1* and the nucleosome remodeling factor *nurf-1*.

While this provides an important foundation upon which to build, the number of direct HIF-1 targets are no doubt much higher than the 96 genes described in S9 Table. Any one binding site will only be occupied some fraction of time. Another limitation is that, in lieu of a reliable HIF-1-specific antibody, we used an epitope-tagged version of HIF-1. While there are good reasons for focusing on the a isoform, it is likely that other isoforms have biological functions as well. With time and improved technologies, additional studies will add to this core dataset.

## Materials and methods

### Strains

The wild-type *C. elegans* used in this study was N2 Bristol. The mutant strains used in this study were listed in S10 Table. All the worms were maintained at 21°C using the standard methods [32].

### Gene expression microarray experiment

Randomized complete block design was followed for the microarray experiment, with three biological replicates treated as three blocks. Each block included eight treatments: N2 wild type, N2 wild type with hypoxia treatment, *hif-1(ia04)* loss-of-function mutants, *hif-1(ia04)* loss-of-function mutants with hypoxia treatment, *vhl-1(ok161)* loss-of-function mutants, *rhy-1(ok1402)* loss-of-function mutants, *egl-9(sa307)* loss-of-function mutants and *swan-1(ok267);vhl-1(ok161)* loss-of-function double mutants. For each treatment, about 1,000 synchronized L4-stage larvae were pooled as one experimental unit to get sufficient RNA for hybridization. Total RNA isolation was performed using Trizol (Invitrogen) and RNeasy Mini Kit (Qiagen). RNA quality was checked with an Agilent 2100 BioAnalyzer (Agilent Technologies). The RNA integrity numbers (RINs) for all the samples used in this study were greater than 9.0. The total RNA isolated from one experimental unit was hybridized onto one Affymetrix GeneChip^®^ C. elegans Genome array (Affymetrix, part number 900383). Probe synthesis, labeling, hybridization, washing, staining and scanning were performed by the GeneChip facility at Iowa State University. In brief, the total RNA was synthesized to biotin-labeled aRNA using the GeneChip^®^ 3’ IVT Express Kit (Affymetrix, part number 901229) and hybridized to the array. The arrays were washed and stained in the GeneChip^®^ fludics station 450 and scanned with GeneChip^®^ scanner 3000 7G. The Affymetrix^®^ GeneChip^®^ Command Console^™^ (AGCC) software was used to generate probe cell intensity data (.CEL) files. The resulting CEL files were normalized and summarized using the robust multichip average (RMA) algorithm [33] in R package (R Core Team, Vienna, Austria, 2016). An analysis of variance (ANOVA) model was then fitted to the summarized expression measures, with the block (three levels) and the treatment (eight levels) treated as fixed effect factors following the experimental design.

Residual model diagnostics identified no severe violations of the model assumptions. Linear contrasts of treatment means were tested using the general F-test. To account for multiplicities of hypothesis testing, conservative estimates of false discovery rates (FDRs) were calculated according to the *q*-value procedure of Storey and Tibshirani [34]. Differentially expressed probesets were defined as *q*-value ≤ 0.05 and fold change ≥ 1.6. Probesets were converted to genes using the Affymetrix annotation file “Celegans.na36.annot.csv. To deal with redundancy and count the number of unique genes detected on the array, we kept one probeset per gene and one gene per probeset. In this way, the total number of unique genes detected on the array was 18, 011. For the purpose of reference, the original complete lists of gene(s) annotated to each probeset were kept in S1-S3, S6 and S7.

In this paper, we discuss HIF-1-dependent hypoxia responses. Gene expression changes in the mutants with constitutive HIF-1 activity will be described in a related study entitled “Transcriptome analyses describe the consequences of persistent HIF-1 over-activation in *Caenorhabditis elegans*”. The microarray raw and probeset summary data had been deposited to NCBI’s Gene Expression Omnibus, the accession number was GSE228851.

### Gene function annotation and enrichment analyses

DAVID tools (The Database for Annotation, Visualization and Integrated Discovery) [35, 36] (https://david.ncifcrf.gov) were used to annotate the enriched biological terms associated with microarray and ChIP-seq-selected genes. The enriched biological terms were at *p*-value < 0.01 with no correction.

### Heat maps

Heat maps for gene expression profiles were generated by the PermutMatrix graphical analysis program [37, 38]. Average linkage clustering was performed using the hypoxia induction values. Green color represented negative values, and red color represented positive values. The intensities of the colors represented the magnitudes of fold changes. Other parameters were set as default.

### Gene lists overlap testing

Fisher’s exact test was performed to test whether the overlap between two gene lists was significant or not. The total number of 18, 011 genes detected on the microarray was used as the population size. The significant overlap is at *p*-value < 0.001.

### HIF-1 chromatin immunoprecipitation sequencing (ChIP-seq)

The ChIP experiments were performed in the *egl-9(sa307)* loss-of-function mutant background to maintain HIF-1 stability and activity in room air. The strain used for the ChIP experiments was ZG434 (*egl-9(sa307);iaIS28[Phif-1::hif-1a::Myc::HA];hif-1(ia04)*). To use the commercially available ChIP grade anti-HA antibody (Abcam, cat. no. ab9110), an HA-tagged *hif-1a* transgene *iaIS28[Phif-1::hif-1a::Myc::HA]*) [25] was introduced into *egl-9(sa307)*, and the endogenous *hif-1* gene was knocked out. Detailed ChIP protocol was provided in S7 file. Briefly, synchronized L4-stage worms for each biological replicate were harvested in separate batches at separate times. Harvesting enough synchronized worms for HIF-1 ChIP experiment was laborious due to the egg-laying defect inherent to the *egl-9(sa307)* loss-of-function mutation. For each batch, about 10, 000 L4-stage worms were harvested and cross-linked in 2% formaldehyde at 21°C for 30 minutes. The ChIP-seq experiment was performed with two biological replicates. For each biological replicate, nuclear lysates from about 50,000 worms (pooled from 5 batches of worm collection) were sonicated using a Branson sonifer microtip to fragment the chromatin to 200-800 bp. Immunoprecipitated protein-DNA complexes were captured on protein A-Sepharose beads (Sigma) and eluted in elution buffer (1% SDS and 100 mM NaHCO3) at 65°C for 30 minutes. Following RNase treatment and cross-link reversal, the ChIP DNA was purified with the Qiagen MinElute Kit and stored at −20°C for sequencing in parallel with the corresponding input DNA. The ChIP-Seq library preparation and sequencing were performed by the Iowa State University DNA facility. In brief, NEXTflex^™^ ChIP-Seq Barcodes kit (Illumina compatible) (BIOO Scientific Corp., cat. no. 514123) was used to prepare multiplexed single-end genomic DNA libraries. The gel slices corresponding to the 200-300 bp maker were cut and purified. The purified DNA was amplified and sequenced in a single flow cell on the IlluminaHiSeq 2000 platform. The length of reads was 50 bp.

### HIF-1 ChIP-seq data analyses

The HIF-1 ChIP-seq raw and processed data had been deposited to NCBI’s Gene Expression Omnibus and the accession number was: GSE228846. The quality scores of the fastq reads for the input and ChIP DNA samples from both biological replicates were all above 30, indicating high quality sequencing data (S7 Fig). Reads were mapped to *C. elegans* reference genome ce11 using bowtie 2 with the default settings. Reads with mapping quality (MAPQ) score less than 10 or reads mapped to the mitochondrial genome were excluded. At the end, reads kept for peak calling were 19-43 million per sample. The kept reads were assigned to bins, the size of which was set at 200 bp to match the fragment length for Illumina sequencing. Bin-level read counts were analyzed by the R package MOSAiCS (MOdel-based one and two Sample Analysis and Inference for ChIP-Seq Data) to call peaks [39] (https://www.bioconductor.org/packages/release/bioc/manuals/mosaics/man/mosaics.pdf). The false discovery rate (FDR) was set at 0.05. Neighboring peaks were merged. The output peaks were further filtered with the following criteria: (1) minimum posterior probability ≤ 0.05; (2) averaged input tag count ≥ 10; (3) averaged ChIP tag count ≥ 10; and (4) fold enrichment (averaged ChIP tag count/normalized average input tag count) ≥ 1.6. The identified peaks were visually verified in IGB (Integrated Genome Browser) [40]. The WIG files for IGB were provided in GEO database (accession number GSE228846). Peaks identified by both biological replicates were treated as HIF-1 binding regions. Peaks were assigned to genes within 6 kb. Within this region, if there was a gene(s) differentially expressed under hypoxia or in the HIF-1 negative regulator mutants (*vhl-1(ok161), egl-9(sa307), rhy-1(ok1402)* and *swan-1(ok267);vhl-1(ok161)* mutants), the peak was assigned to this gene. We reasoned that a gene(s) showed expression change under these conditions was more possible to be a HIF-1 direct target than genes showed no expression changes. Otherwise the nearest gene was assigned to the peak. Most often (90 out of 96 genes), the assigned HIF-1 direct target was the nearest gene.

### ChIP-qPCR to verify the HIF-1 binding site in the *efk-1* promoter

The primers for ChIP-qPCR to verify the HIF-1 binding region in the *efk-1* promoter were the forward primer 5’-CAATCTGACCGAGCCGAATG-3’ and reverse primer 5’-AGGCCTTTCTCGATTTTCCA-3’. The amplicon was 172 bp and contained a HRE 5’-ACGTG-3’. The promoter region of *sir-2*, a gene not regulated by HIF-1 under short-term hypoxia or in the HIF-1 negative regulator mutants, was used as the reference. The primers for *sir-2* ChIP-qPCR were the forward primer 5’-AGATTGCTTCTTTGGCTGGA-3’ and reverse primer 5’-GTAACGCACCTTGCAACAGA-3’. The amplicon was 218 bp and did not contain HRE similar sequences. Three biological replicates were performed. qPCR quantification was performed using the efficiency-corrected comparative quantification method [41].

### Hypoxia development and survival assays

For each mutant genotype, the room air and hypoxia treatments were performed in parallel at 21°C. For each treatment, 20 young adults (one day after L4 molt) were used as parents to lay eggs on one NGM plate seeded with OP50 for 30 minutes. After counting the eggs laid, the plates were kept in room air or put into a sealed plexiglass chamber with constant hypoxic gas flow for 24 hours. Compressed air and 100% nitrogen were mixed to achieve 0.5% oxygen, and gas flow was controlled by an oxygen sensor [18]. After 24 hours, the un-hatched eggs were counted for both treatments. After that, the plates for both treatments were maintained in room air. The adult worms were counted 72 hours after the eggs had been laid. The data collection time points were set to match the development rate of N2 eggs in room air: they hatched within 24 hours and reached adulthood within 72 hours.

For RNAi strains, RNAi was induced by bacterial feeding as described [42, 43]. Except for F57B9.1, *gbh-2* and *comt-4*, the RNAi clones were purchased from the Ahringer RNAi library (Geneservice, Cambridge, UK) and validated by sequencing. The RNAi constructs for F57B9.1, *gbh-2* and *comt-4* were generated by cloning the coding regions into the L4440 double-T7 vector [42]. To generate the RNAi parent generation, 20 N2 adults maintained with OP50 were transferred to RNAi plates to lay eggs for 1 hour. Three days later, 20 young adults grown up from these eggs were randomly picked as RNAi parents to lay eggs on a new RNAi plate for 30 minutes for either room air or hypoxia treatment. The room air and hypoxia treatments were performed in parallel at 21°C. The downstream procedures for hypoxia treatment and counting the hatched/un-hatched eggs and adult/non adult animals were the same as those described above for the mutant strains.

The experiments were performed with three biological replicates. To test the effect of hypoxia on animal development and survival, the binary hatched vs. un-hatched or adult *vs*. non adult data were analyzed by fitting a generalized linear model using a logit link function with JMP 9 statistical software (SAS Institute Inc., Cary, NC, 2010). The replicate (three levels) and the treatment (two levels) were used as factors in the model. For situations in which such models were inappropriate, randomization tests were used.

## Supplementary Material

1

## Figures and Tables

**Table 1.** Enriched biological terms for genes up-regulated by short-term hypoxia in N2.

Biological term	Count	%	*p*-value	Representative genes
Oxidation-reduction process	51	12	3.74E-14	*fmo-1, fmo-2, cyp-14A2, egl-9, hgo-1, sqrd-1, phy-2*
Response to stress	66	15	8.37E-11	*hsp-70, cyp-37B1, cdr-2, nhr-115, ctl-1, mtl-1, ugt-44, nhr-57*
Transition metal ion binding	62	14	1.07E-10	*nhr-228, cyp-37B1, nurf-1, egl-9, mtl-1*
Small molecule metabolic process	43	10	2.64E-07	*cysl-3, pck-1, ugt-17, mmaa-1, mai-1, cysl-2*
Cellular response to unfolded protein	13	3	1.21E-06	*hsp-16.1, xbp-1, pek-1*

**Table 2.** Enriched biological terms for genes down-regulated by short-term hypoxia in N2.

Biological term	Count	%	*p*-value	Representative genes
Single-organism metabolic process	73	30	1.75E-17	*ugt-31, acox-3, cyp-25A1, sur-5, fat-7, gcsh-1, lipl-1*
Monocarboxylic acid metabolic process	21	9	1.02E-10	*ugt-12, fat-7, acox-3, elo-2, hacd-1*
Oxidation-reduction process	29	12	1.63E-07	*fat-7, gst-10, cyp-29A2, fard-1, gpdh-1*

**Table 3.** Enriched biological terms for genes positively regulated by HIF-1 under short-term hypoxia.

Biological term	Count	%	*p*-value	Genes
Oxidation-reduction process	17	27	4.39E-10	F41E6.5, ZK550.6, F57B9.1, Y105C5B.9, *fmo-1, fmo-2, fat-5, egl-9, hgo-1, sqrd-1, gbh-2, acox-1.6*/F59F4.1, F21D5.3, *ddo-1, phy-2, stdh-1, pah-1*
Metabolic pathways	11	17	9.35E-03	*acox-1.6/*F59F4.1, *asns-2, cysl-2*, F41E6.5, F57B9.1, *hgo-1, mce-1, mmcm-1, pah-1, pck-1, phy-2*
Alpha-amino acid metabolic process	5	8	5.04E-04	*asns-2, pah-1, cysl-2, egl-9, hgo-1*
Fatty acid metabolic process	5	8	6.87E-04	*acox-1.6*/F59F4.1, F58A6.1, ZK550.6, *fat-5*, C32E8.9
Sulfur metabolism	3	5	2.91E-03	*cysl-2, ethe-1, sqrd-1*

**Table 4.** Enriched biological terms for genes negatively regulated by HIF-1 under short-term hypoxia.

Biological term	Count	%	*p*-value	Genes
Response to stress	22	37	1.42E-10	C49G7.10, *ckb-2, elo-2*, F13A7.11, F18H3.4, F41B4.3, F56D2.5, *fbxa-60, fbxc-58, ipla-2, irg-2*, K09D9.1, *nipi-3, parg-2, skr-3*, T24C4.4, *tir-1, tmem-135, ugt-44, warf-1*, Y41C4A.11, Y43F8B.2
Organic acid metabolic process	9	15	1.25E-04	*acdh-2, acs-2, ddo-2, elo-2, hacd-1, sur-5, ugt-25, ugt-28, ugt-44*

**Table 5.** Hypoxia responses of genes positively regulated by both short-term and persistent HIF-1 activities.

Probeset ID	Gene	Description	Hypoxia induction in N2	Hypoxia induction in *hif-1*	Relative induction (N2/*hif-1*)	*q*-value
175837_s_at	*efk-1*	Elongation factor-2 kinase	3.16	1.31	2.40	2.31E-02
187973_s_at	*egl-9*	Dioxygenase	3.41	1.04	3.27	7.05E-04
191695_at	*mce-1*	Methylmalonyl-CoA epimerase	2.32	1.05	2.22	7.31E-05
191736_s_at	*mmcm-1*	Methylmalonyl-CoA mutase	1.69	−1.03	1.74	1.45E-02
177532_at	F22B5.4	Integral membrane protein	3.46	−1.04	3.61	3.53E-03
188978_s_at	K10H10.2/*cysl-2*	Cysteine synthase	3.28	−1.43	4.69	1.47E-03
180842_s_at	*ethe-1*	Sulfur dioxygenase	3.51	1.54	2.27	5.46E-05
178043_at	*sqrd-1*	Sulfide quinone oxidoreductase	8.55	1.15	7.43	3.36E-06
173335_s_at	*dod-3*	Downstream Of DAF-16 (regulated by DAF-16)	57.42	20.34	2.82	1.65E-02
174073_at	*tts-1*	Transcribed telomere-like sequence	93.70	23.24	4.03	2.60E-02
193464_s_at	*phy-2*	Collagen prolyl 4-hydroxylase	7.44	−1.10	8.22	7.38E-05
193914_at	*gbh-2*	Gamma butyrobetaine hydroxylase	3.23	−1.21	3.91	1.36E-04
190962_s_at	*gst-19*	Glutathione S-transferase	1.85	−1.18	2.19	9.13E-03
192338_at	*hsp-12.3*	Heat shock protein	2.85	−1.06	3.01	8.99E-03
180909_at	C32H11.4	Hypothetical protein	2.55	−1.59	4.06	1.40E-03
182532_at	*mpst-3*	Mercaptopyruvate sulfurtransferase homolog	1.24	−1.44	1.79	1.66E-03
182849_at	Y37A1B.5	Selenium binding protein	3.04	1.03	2.95	1.97E-04
186811_s_at	ZK550.6	Peroxisomal phytanoyl-CoA hydroxylase	4.26	−1.02	4.35	2.01E-05
173475_s_at	*glb-1*	Globin	3.07	−1.19	3.66	2.24E-05
186801_s_at	F45D11.14	Hypothetical protein	35.75	4.87	7.34	1.33E-03
182747_at	R08E5.3	Methyltransferase	3.03	1.43	2.12	2.96E-02
192581_s_at	W05G11.6/*pck-1*	Phosphoenolpyruvate carboxykinase (PEPCK)	3.10	−1.41	4.36	1.03E-02
184714_s_at	Y53G8B.2	2-acylglycerol O-acyltransferase	3.16	1.29	2.46	4.75E-04

**Table 6.** Hypoxia responses of genes negatively regulated by both short-term and persistent HIF-1 activities.

Probeset ID	Gene	Description	Hypoxia-induction in N2	Hypoxia induction in *hif-1*	Relative induction (N2/*hif-1*)	*q*-value
184849_at	T28A11.2	Hypothetical protein	−1.28	1.47	−1.88	1.28E-02
173996_at	*acdh-2*	Acyl CoA dehydrogenase	1.05	3.22	−3.08	1.17E-02
174675_at	*acs-2*	Fatty acid CoA synthetase family	5.29	16.74	−3.17	1.07E-02

**Table 7.** Enriched biological terms associated with HIF-1 direct target genes.

Biological term	Count	%	*p*-value	Genes
Protein processing in endoplasmic reticulum	7	7.29	5.74E-06	*dnj-12, ero-1*, F44E5.4, F44E5.5, *hsp-70, hsp-90, unc-23*
Response to stress	14	14.58	4.36E-04	*atg-5, cpr-3, daf-25, dnj-12, ero-1, hsp-16.2, hsp-16.41, hsp-70, hsp-90, hsp-110, irg-1, tir-1, tank-1*, Y56A3A.33
Cell recognition	4	4.17	5.85E-03	*cua-1, madd-2*, Y105E8B.9, ZC262.7

**Table 8.** Genes that co-immunoprecipitated with HIF-1 and were shown to be regulated by HIF-1.

Expression	Count	Genes
Positively regulated by HIF-1 under short-term hypoxia	3	*efk-1, sqrd-1*, W03F9.1
Negatively regulated by HIF-1 under short-term hypoxia	1	*tir-1*
Up-regulated in at least one mutant that stabilizes HIF-1	14	Y105E8B.9, F44E5.5, F44E5.4, *nurf-1, efk-1*, C46C2.5, *sqrd-1, hsp-16.41, hsp-16.2, irg-1, ari-1.4/tag-349*, F54D5.12, *nurf-1, cpr-3*
Down-regulated in at least one mutant that stabilizes HIF-1	9	C50A2.3, C46C2.5, F54D5.12, *oat-1*, Y105C5B.5, F19B2.5, M04F3.3/*kin-35*, Y94H6A.5, C23H5.8

## References

[1] Semenza GL. Hypoxia-inducible factors in physiology and medicine. Cell. 2012;148(3):399–408. Epub 2012/02/07. doi: S0092-8674(12)00087-6 [pii] 10.1016/j.cell.2012.01.021. 22304911 PMC3437543

[2] Wang GL, Jiang BH, Rue EA, Semenza GL. Hypoxia-inducible factor 1 is a basic-helix-loop-helix-PAS heterodimer regulated by cellular O2 tension. Proc Natl Acad Sci U S A. 1995;92(12):5510–4. Epub 1995/06/06. 7539918 10.1073/pnas.92.12.5510 PMC41725

[3] Takahata S, Sogawa K, Kobayashi A, Ema M, Mimura J, Ozaki N, Transcriptionally active heterodimer formation of an Arnt-like PAS protein, Arnt3, with HIF-1a, HLF, and clock. Biochemical and biophysical research communications. 1998;248(3):789–94. Epub 1998/08/15. doi: 10.1006/bbrc.1998.9012. 9704006

[4] Semenza GL, Jiang BH, Leung SW, Passantino R, Concordet JP, Maire P, Hypoxia response elements in the aldolase A, enolase 1, and lactate dehydrogenase A gene promoters contain essential binding sites for hypoxia-inducible factor 1. J Biol Chem. 1996;271(51):32529–37. Epub 1996/12/20. 8955077 10.1074/jbc.271.51.32529

[5] Kaelin WG Jr., Ratcliffe PJ. Oxygen sensing by metazoans: the central role of the HIF hydroxylase pathway. Mol Cell. 2008;30(4):393–402. Epub 2008/05/24. doi: S1097-2765(08)00292-X [pii] 10.1016/j.molcel.2008.04.009. 18498744

[6] Berra E, Ginouves A, Pouyssegur J. The hypoxia-inducible-factor hydroxylases bring fresh air into hypoxia signalling. EMBO Rep. 2006;7(1):41–5. Epub 2006/01/05. doi: 7400598 [pii] 10.1038/sj.embor.7400598. 16391536 PMC1369233

[7] Powell-Coffman JA. Hypoxia signaling and resistance in C. elegans. Trends Endocrinol Metab. 2010;21(7):435–40. Epub 2010/03/26. doi: S1043-2760(10)00041-X [pii] 10.1016/j.tem.2010.02.006. 20335046

[8] Iyer NV, Kotch LE, Agani F, Leung SW, Laughner E, Wenger RH, Cellular and developmental control of O2 homeostasis by hypoxia-inducible factor 1 alpha. Genes Dev. 1998;12(2):149–62. Epub 1998/03/07. 9436976 10.1101/gad.12.2.149 PMC316445

[9] Ryan HE, Lo J, Johnson RS. HIF-1 alpha is required for solid tumor formation and embryonic vascularization. EMBO J. 1998;17(11):3005–15. Epub 1998/06/26. doi: 10.1093/emboj/17.11.3005. 9606183 PMC1170640

[10] Padilla PA, Nystul TG, Zager RA, Johnson AC, Roth MB. Dephosphorylation of cell cycle-regulated proteins correlates with anoxia-induced suspended animation in Caenorhabditis elegans. Mol Biol Cell. 2002;13(5):1473–83. Epub 2002/05/15. doi: 10.1091/mbc.01-12-0594. 12006646 PMC111120

[11] Jiang H, Guo R, Powell-Coffman JA. The Caenorhabditis elegans hif-1 gene encodes a bHLH-PAS protein that is required for adaptation to hypoxia. Proc Natl Acad Sci U S A. 2001;98(14):7916–21. Epub 2001/06/28. doi: 10.1073/pnas.141234698 141234698 [pii]. 11427734 PMC35443

[12] Nystul TG, Roth MB. Carbon monoxide-induced suspended animation protects against hypoxic damage in Caenorhabditis elegans. Proc Natl Acad Sci U S A. 2004;101(24):9133–6. doi: 10.1073/pnas.0403312101. 15184665 PMC428485

[13] Epstein AC, Gleadle JM, McNeill LA, Hewitson KS, O’Rourke J, Mole DR, C. elegans EGL-9 and mammalian homologs define a family of dioxygenases that regulate HIF by prolyl hydroxylation. Cell. 2001;107(1):43–54. Epub 2001/10/12. doi: S0092-8674(01)00507-4 [pii]. 11595184 10.1016/s0092-8674(01)00507-4

[14] Powell-Coffman JA, Bradfield CA, Wood WB. Caenorhabditis elegans orthologs of the aryl hydrocarbon receptor and its heterodimerization partner the aryl hydrocarbon receptor nuclear translocator. Proc Natl Acad Sci U S A. 1998;95(6):2844–9. Epub 1998/04/18. 9501178 10.1073/pnas.95.6.2844 PMC19657

[15] Shao Z, Zhang Y, Powell-Coffman JA. Two Distinct Roles for EGL-9 in the Regulation of HIF-1-Mediated Gene Expression in Caenorhabditis elegans. Genetics. 2009;183:821–9. Epub 2009/09/10. doi: genetics.109.107284 [pii] 10.1534/genetics.109.107284. 19737748 PMC2778979

[16] Shen C, Shao Z, Powell-Coffman JA. The Caenorhabditis elegans rhy-1 gene inhibits HIF-1 hypoxia-inducible factor activity in a negative feedback loop that does not include vhl-1. Genetics. 2006;174(3):1205–14. Epub 2006/09/19. doi: genetics.106.063594 [pii] 10.1534/genetics.106.063594. 16980385 PMC1667075

[17] Doering KRS, Cheng X, Milburn L, Ratnappan R, Ghazi A, Miller DL, Nuclear hormone receptor NHR-49 acts in parallel with HIF-1 to promote hypoxia adaptation in Caenorhabditis elegans. eLife. 2022;11. doi: 10.7554/eLife.67911. PMC8959602 35285794

[18] Shen C, Nettleton D, Jiang M, Kim SK, Powell-Coffman JA. Roles of the HIF-1 hypoxia-inducible factor during hypoxia response in Caenorhabditis elegans. J Biol Chem. 2005;280(21):20580–8. Epub 2005/03/23. doi: M501894200 [pii] 10.1074/jbc.M501894200. 15781453

[19] Bishop T, Lau KW, Epstein AC, Kim SK, Jiang M, O’Rourke D, Genetic analysis of pathways regulated by the von Hippel-Lindau tumor suppressor in Caenorhabditis elegans. PLoS Biol. 2004;2(10):e289. Epub 2004/09/14. doi: 10.1371/journal.pbio.0020289. 15361934 PMC515368

[20] Vora M, Pyonteck SM, Popovitchenko T, Matlack TL, Prashar A, Kane NS, The hypoxia response pathway promotes PEP carboxykinase and gluconeogenesis in C. elegans. Nat Commun. 2022;13(1):6168. doi: 10.1038/s41467-022-33849-x. 36257965 PMC9579151

[21] Budde MW, Roth MB. The response of Caenorhabditis elegans to hydrogen sulfide and hydrogen cyanide. Genetics. 2011;189(2):521–32. Epub 2011/08/16. doi: genetics.111.129841 [pii] 10.1534/genetics.111.129841. 21840852 PMC3189795

[22] Horsman JW, Miller DL. Mitochondrial Sulfide Quinone Oxidoreductase Prevents Activation of the Unfolded Protein Response in Hydrogen Sulfide. J Biol Chem. 2016;291(10):5320–5. doi: 10.1074/jbc.M115.697102. 26677221 PMC4777863

[23] Harris TW, Antoshechkin I, Bieri T, Blasiar D, Chan J, Chen WJ, WormBase: a comprehensive resource for nematode research. Nucleic Acids Res. 2010;38(Database issue):D463–7. Epub 2009/11/17. doi: 10.1093/nar/gkp952. 19910365 PMC2808986

[24] Myllyharju J. Prolyl 4-hydroxylases, key enzymes in the synthesis of collagens and regulation of the response to hypoxia, and their roles as treatment targets. Ann Med. 2008;40(6):402–17. doi: 10.1080/07853890801986594. 19160570

[25] Zhang Y, Shao Z, Zhai Z, Shen C, Powell-Coffman JA. The HIF-1 hypoxia-inducible factor modulates lifespan in C. elegans. PLoS One. 2009;4(7):e6348. Epub 2009/07/28. doi: 10.1371/journal.pone.0006348. 19633713 PMC2711329

[26] Luhachack LG, Visvikis O, Wollenberg AC, Lacy-Hulbert A, Stuart LM, Irazoqui JE. EGL-9 controls C. elegans host defense specificity through prolyl hydroxylation-dependent and - independent HIF-1 pathways. PLoS Pathog. 2012;8(7):e1002798. Epub 2012/07/14. doi: 10.1371/journal.ppat.1002798. 22792069 PMC3390412

[27] Kudron MM, Victorsen A, Gevirtzman L, Hillier LW, Fisher WW, Vafeados D, The ModERN Resource: Genome-Wide Binding Profiles for Hundreds of Drosophila and Caenorhabditis elegans Transcription Factors. Genetics. 2018;208(3):937–49. doi: 10.1534/genetics.117.300657. 29284660 PMC5844342

[28] Wenger RH, Stiehl DP, Camenisch G. Integration of oxygen signaling at the consensus HRE. Sci STKE. 2005;2005(306):re12. Epub 2005/10/20. doi: stke.3062005re12 [pii] 10.1126/stke.3062005re12. 16234508

[29] Murphy CT, McCarroll SA, Bargmann CI, Fraser A, Kamath RS, Ahringer J, Genes that act downstream of DAF-16 to influence the lifespan of Caenorhabditis elegans. Nature. 2003;424(6946):277–83. Epub 2003/07/08. doi: 10.1038/nature01789 [pii]. 12845331

[30] Miller DL, Budde MW, Roth MB. HIF-1 and SKN-1 coordinate the transcriptional response to hydrogen sulfide in Caenorhabditis elegans. PLoS One. 2011;6(9):e25476. Epub 2011/10/08. doi: 10.1371/journal.pone.0025476 PONE-D-11-09953 [pii]. 21980473 PMC3183046

[31] Félix M-A, Braendle C. The natural history of Caenorhabditis elegans. Current Biology. 2010;20(22):R965–R9. doi: 10.1016/j.cub.2010.09.050. 21093785

[32] Brenner S. The genetics of Caenorhabditis elegans. Genetics. 1974;77(1):71–94. Epub 1974/05/01. 4366476 10.1093/genetics/77.1.71 PMC1213120

[33] Irizarry RA, Bolstad BM, Collin F, Cope LM, Hobbs B, Speed TP. Summaries of Affymetrix GeneChip probe level data. Nucleic Acids Res. 2003;31(4):e15. Epub 2003/02/13. 12582260 10.1093/nar/gng015 PMC150247

[34] Storey JD, Tibshirani R. Statistical significance for genomewide studies. Proc Natl Acad Sci U S A. 2003;100(16):9440–5. Epub 2003/07/29. doi: 10.1073/pnas.1530509100 1530509100 [pii]. 12883005 PMC170937

[35] Huang da W, Sherman BT, Lempicki RA. Systematic and integrative analysis of large gene lists using DAVID bioinformatics resources. Nat Protoc. 2009;4(1):44–57. doi: 10.1038/nprot.2008.211. 19131956

[36] Huang da W, Sherman BT, Lempicki RA. Bioinformatics enrichment tools: paths toward the comprehensive functional analysis of large gene lists. Nucleic Acids Res. 2009;37(1):1–13. doi: 10.1093/nar/gkn923. 19033363 PMC2615629

[37] Caraux G, Pinloche S. PermutMatrix: a graphical environment to arrange gene expression profiles in optimal linear order. Bioinformatics. 2005;21(7):1280–1. Epub 2004/11/18. doi: bti141 [pii] 10.1093/bioinformatics/bti141. 15546938

[38] Eisen MB, Spellman PT, Brown PO, Botstein D. Cluster analysis and display of genome-wide expression patterns. Proc Natl Acad Sci U S A. 1998;95(25):14863–8. Epub 1998/12/09. 9843981 10.1073/pnas.95.25.14863 PMC24541

[39] Kuan PF, Chung DJ, Pan GJ, Thomson JA, Stewart R, Keles S. A Statistical Framework for the Analysis of ChIP-Seq Data. J Am Stat Assoc. 2011;106(495):891–903. doi: DOI 10.1198/jasa.2011.ap09706. 26478641 PMC4608541

[40] Nicol JW, Helt GA, Blanchard SG Jr., Raja A, Loraine AE. The Integrated Genome Browser: free software for distribution and exploration of genome-scale datasets. Bioinformatics. 2009;25(20):2730–1. Epub 2009/08/06. doi: 10.1093/bioinformatics/btp472 btp472 [pii]. 19654113 PMC2759552

[41] Pfaffl MW. A new mathematical model for relative quantification in real-time RT-PCR. Nucleic Acids Res. 2001;29(9):e45. Epub 2001/05/09. 11328886 10.1093/nar/29.9.e45 PMC55695

[42] Timmons L, Court DL, Fire A. Ingestion of bacterially expressed dsRNAs can produce specific and potent genetic interference in Caenorhabditis elegans. Gene. 2001;263(1-2):103–12. Epub 2001/02/27. doi: S0378111900005795 [pii]. 11223248 10.1016/s0378-1119(00)00579-5

[43] Kamath RS, Fraser AG, Dong Y, Poulin G, Durbin R, Gotta M, Systematic functional analysis of the Caenorhabditis elegans genome using RNAi. Nature. 2003;421(6920):231–7. Epub 2003/01/17. doi: 10.1038/nature01278 nature01278 [pii]. 12529635

