# Supplementary material for: Whole genome profiling of short-term hypoxia induced genes and identification of HIF-1 binding sites provide insights into HIF-1 function in Caenorhabditis elegans
Source: bioRxiv. 2023 Nov 17:2023.11.15.567310. Preprint. [Version 1] doi: 10.1101/2023.11.15.567310 (PMC10680714; doi:10.1101/2023.11.15.567310)
Supplement: 1 [file NIHPP2023.11.15.567310v1-supplement-1.pdf]

bioRxiv preprint doi: https://doi.org/10.1101/2023.11.15.567310; this version posted November 17, 2023. The copyright holder for this preprint (which was not certified by peer review) is the author/funder. This article is a US Government work. It is not subject to copyright under 17 USC 105 and is also made available for use under a CC0 license.

Supporting information

S1 Table. Gene expression for all the probesets under hypoxia and in the HIF-1 negative regulator mutants.

S2 Table. Genes up-regulated by short-term hypoxia in N2.

S3 Table. Genes down-regulated by short-term hypoxia in N2.

S4 Table. Enriched biological terms for genes up-regulated by short-term hypoxia in N2.

S5 Table. Enriched biological terms for genes down-regulated by short-term hypoxia in N2.

bioRxiv preprint doi: https://doi.org/10.1101/2023.11.15.567310; this version posted November 17, 2023. The copyright holder for this preprint (which was not certified by peer review) is the author/funder. This article is a US Government work. It is not subject to copyright under 17 USC 105 and is also made available for use under a CC0 license.

S6 Table. Genes positively regulated by HIF-1 under short-term hypoxia.

S7 Table. Genes negatively regulated by HIF-1 under short-term hypoxia.

S8 Table. Effects of HIF-1-dependent hypoxia responsive genes on hypoxia development and survival.

S9 Table. Direct targets for HIF-1 identified by ChIP-seq.

S10 Table. Descriptions of mutations used in this study.

S1 File. Sequences co-immunoprecipitated with HIF-1 on chromosome 1.

S2 File. Sequences co-immunoprecipitated with HIF-1 on chromosome 2.

S3 File. Sequences co-immunoprecipitated with HIF-1 on chromosome 3.

S4 File. Sequences co-immunoprecipitated with HIF-1 on chromosome 4.

S5 File. Sequences co-immunoprecipitated with HIF-1 on chromosome 5.

S6 File. Sequences co-immunoprecipitated with HIF-1 on chromosome X.

S7 File. Detailed protocol for HIF-1 chromatin immunoprecipitation (ChIP).

S1 Fig. HIF-1 direct targets ChIP-seq IGB signals on Chromosome 1.

S2 Fig. HIF-1 direct targets ChIP-seq IGB signals on Chromosome 2.

S3 Fig. HIF-1 direct targets ChIP-seq IGB signals on Chromosome 3.

S4 Fig. HIF-1 direct targets ChIP-seq IGB signals on Chromosome 4.

S5 Fig. HIF-1 direct targets ChIP-seq IGB signals on Chromosome 5.

S6 Fig. HIF-1 direct targets ChIP-seq IGB signals on Chromosome X.

S7 Fig. Quality scores of fastq reads for the input and ChIP DNA samples.
